# Multiple Quartz Crystals Connected in Parallel for High-Resolution Sensing of Capacitance Changes

**DOI:** 10.3390/s22135030

**Published:** 2022-07-03

**Authors:** Vojko Matko

**Affiliations:** Faculty of Electrical Engineering and Computer Science, University of Maribor, Koroška c. 46, 2000 Maribor, Slovenia; vojko.matko@um.si; Tel.: +386-2-220-7111

**Keywords:** multiple quartz crystals in parallel, quartz capacitive sensing method, temperature compensation, switching mode method

## Abstract

We present a new highly sensitive, low-value capacitance sensor method that uses multiple quartz crystals connected in parallel inside the oscillator. In the experimental setup, the measured (sensible) reactance (capacitance) is connected in parallel to the total shunt capacitance of the quartz crystals, oscillating in the oscillator. Because AT-cut crystals have a certain nonlinear frequency–temperature dependence, we use the switching mode method, by which we achieve a temperature compensation of the AT-cut crystals’ frequency–temperature characteristics in the temperature range between $0–{50\;}^{{}^{\circ}}C$. The oscillator switching method also compensates for any other influences on the frequency of the oscillator, such as ageing of the crystals and oscillator elements, supply voltage fluctuations, and other parasitic impedances in the oscillating circuit. Subsequently using two 50-ms-delayed switches between the measuring and reference capacitors, the experimental error in measuring the capacitance is lowered for measurements under a dynamic temperature variation in the range of $0–{50\;}^{{}^{\circ}}C$. The experimental results show that the switching method, which includes a multiple quartz connection and high-temperature compensation improvement of the quartz crystals’ characteristics, enables a sub-aF resolution. It converts capacitance changes in the range 10 zF–200 fF to frequencies in the range 4 kHz–100 kHz.

## 1. Introduction

The use of a single quartz crystal for sensing is a well-known and important measuring technique based on the stability and reliability of the crystal oscillation [1,2,3,4]. Next to the mechanical influence on the crystal oscillation, one can also affect the reactance of the crystal and thus its serial resonant frequency [5,6,7,8,9]. Because the change in frequency is measurable already for extremely low changes in the reactance (for capacitance changes in the region of aF and zF), the method is useful for several applications, such as mechanical displacement, nanopositioning, eccentric motion, strain sensing, dielectric properties of liquids, density of liquids, small volumes, low pressure, etc. [10,11,12,13,14,15,16,17,18]. In addition, when the reactance-to-frequency conversion is used, quartz crystals are applicable as biosensors, in medicine and at specific chemical measurements [14,19,20,21,22,23,24]. The reactance is first transformed into a frequency signal and then into a measured physical or chemical quantity. The reactance-to-frequency conversion is important in measurements where tiny changes in the capacitance (in the region of aF and zF) have to be measured with a high accuracy [3,10,19,23].

The basic purpose of the research presented in this paper was to improve the sensitivity of the high-resolution reactance-to-frequency transducer used in previous research [5,25] by connecting several quartz crystals in parallel in the oscillator. The research is oriented towards a comparison of oscillators with one, two, or three quartz crystals connected in parallel, when the load capacitor (capacitive reactance) with capacitance of the order fF is connected in parallel with the quartz crystals. Because quartz crystals have their own frequency–temperature characteristic that depends on the cut angle, we use the switching mode operation of the oscillator for temperature compensation.

Compared to other methods [26,27,28,29], the major advantageous characteristics of the proposed method are as follows: a conversion of reactance into frequency (measuring range in the fF region with sensitivity in the zF region); compensation of the quartz eigen temperature characteristics; and a simultaneous temperature compensation by the reference frequency oscillator [30]. By the proposed method, we also compensate for the ageing of the crystals [31], the effect of the reference capacity and parasitic impedances in the circuit [22,32], and the effects of nonideal properties of the electronic components, which affect the oscillation frequency. Important also is the compensation of small changes in the supply voltage of the oscillator, which, unless compensated, affects the stability of the oscillator frequency [27,33,34,35,36]. An important advantage of the switching method is also a high temperature dynamic stability and an extended temperature region of operation ($0–{50\;}^{{}^{\circ}}C$). The switching mode operation of the oscillator enables a high stability of oscillation at a very low sensing capacity and reduces the start-up time from ≅20 min to ≅1 min [33,37,38]. The method is simple with a low number of elements that are required in the circuit. In addition, crystals with different cut angles can be used for different applications.

The paper is structured as follows. First, we present an equivalent circuit of a quartz crystal and study the impedance circles if shunt capacitors are connected in parallel to one, two, or three quartz crystals, also connected in parallel. Then we study the resonant conditions for different combinations of crystals and shunt capacitances. Next, we present the experimental setup, the results of the measurements, and finally discuss the obtained results.

## 2. Impedance of One, Two, and Three Quartz Crystals in Parallel

The series resonance frequency $f_{0}$ of a single AT-quartz crystal is

(1) $$f_{0}=\frac{1}{2\pi \sqrt{LC}\;},\;$$

where L and C are motional inductance and capacitance, respectively. A quartz crystal is represented by an equivalent circuit in which a coil with inductance L, capacitor with inductance C, and resistor with a motional resistance R are connected in series, while a shunt capacitor with capacitance $C_{0}$ is connected in parallel [7,8,9,34,37].

A complex impedance ($\bar{Z}$) of one quartz crystal is given as

(2) $$\bar{Z}=\frac{\left(R+j\omega L+\frac{1}{j\omega C}\right)\frac{1}{j\omega C_{0}}}{R+j\omega L+\frac{1}{j\omega C}+\frac{1}{j\omega C_{0}}}\;,\;$$

where ω is the angular frequency and j is the imaginary unit. The shunt capacitance $C_{0}$ in Equation (2) presents the capacitance between the electrodes and a stray capacitance associated with the mounting structure.

To study the impedance close to the resonant frequency, we define the frequency ratio ($\Omega_{r}$)

(3) $$\Omega_{r}=\frac{\omega }{\omega_{0}}\;,\;$$

where $\omega_{0}=1/\sqrt{LC}$ is the resonant angular frequency. Equation (2) can now be transformed into [7]:

(4) $$\bar{Z}\left(\Omega_{r}\right)=R\frac{1+j\frac{\omega_{0}L}{R}\left(\Omega_{r}-\frac{1}{\Omega_{r}}\right)}{1+\frac{C_{0}}{C}\left(1-\Omega_{r}{}^{2}\right)+j\frac{C_{0}}{C}\frac{R}{\omega_{0}L}\Omega_{r}}\;.\;$$

For two crystals in parallel, the complex impedance (${\bar{Z}}_{2Q}\left(\Omega_{r}\right)$) is

(5) $${\bar{Z}}_{2Q}\left(\Omega_{r}\right)=\frac{{\bar{Z}}_{1}\left(\Omega_{r}\right)\;{\bar{Z}}_{2}\left(\Omega_{r}\right)}{{\bar{Z}}_{1}\left(\Omega_{r}\right)+{\bar{Z}}_{2}\left(\Omega_{r}\right)}\;,\;$$

where indices 1 and 2 denote the first and the second quartz crystal, respectively. In the case of two identical crystals, Equation (5) reduces to ${\bar{Z}}_{2Q}\left(\Omega_{r}\right)=\bar{Z}\left(\Omega_{r}\right)/2$, where $\bar{Z}\left(\Omega_{r}\right)$ is given by Equation (4).

**Figure 1:**
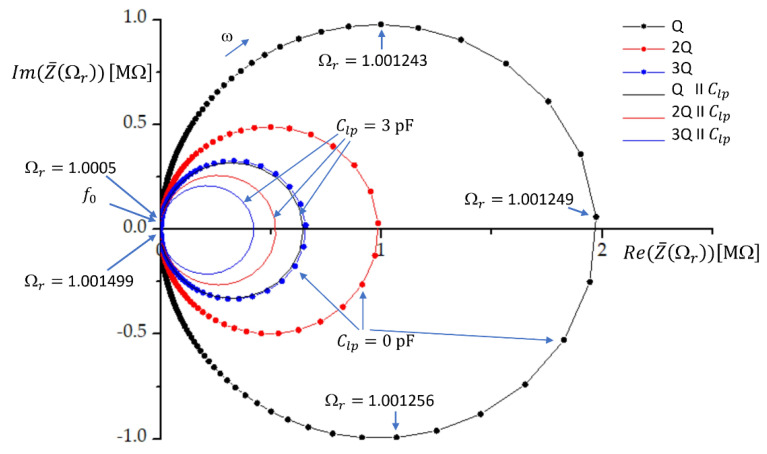
The impedance curves for one, two, and three quartz crystals connected in parallel without (line and symbols) and with (symbols) the load capacitor with capacitance $C_{lp}$ in parallel. $\Omega_{r}$ is the frequency ratio. The quartz crystal parameter values are C=10 fF, L=158.314 mH, $C_{0}=4\;\mathrm{pF}$, R=10  Ω, and $f_{0}=4\;\mathrm{MHz}$.

Three crystals in parallel can be considered as one crystal in parallel with a crystal dublet, and from Equation (5) we deduce the expression for the complex impedance (${\bar{Z}}_{3Q}\left(\Omega_{r}\right)$) of a triplet:

(6) $${\bar{Z}}_{3Q}\left(\Omega_{r}\right)=\frac{{\bar{Z}}_{2Q}\left(\Omega_{r}\right)\;{\bar{Z}}_{3}\left(\Omega_{r}\right)}{{\bar{Z}}_{2Q}\left(\Omega_{r}\right)+{\bar{Z}}_{3}\left(\Omega_{r}\right)}\;,\;$$

where index 3 denotes the third quartz crystal. In the case of three identical crystals, Equation (6) reduces to ${\bar{Z}}_{3Q}\left(\Omega_{r}\right)=\bar{Z}\left(\Omega_{r}\right)/3$. The impedance characteristics for one, two, and three quartz crystals in parallel are given in Figure 1. The ratio $\Omega_{r}$ varied from 1.0005 to 1.001499 with a step of $10^{-6}$. The quartz crystal parameters chosen for plotting were C=10 fF, L=158.314 mH, $C_{0}=4\;\mathrm{pF}$, and R=10 Ω. At these parameters, the resonant frequency is $f_{0}=4\;\mathrm{MHz}$. At the resonant frequency $f_{0}$, the real and imaginary parts of the impedance are very low. When the (angular) frequency increases, the impedance curve crosses the real axis; thus, the imaginary part of the impedance is zero at a very high real part of impedance. This frequency is called the antiresonance or parallel resonance frequency of the crystal and depends on the value of the shunt capacitance. $\Omega_{r}$ at the parallel resonance frequency is given by $\Omega_{r}=\sqrt{1+C/C_{0}}$ [7].

In this research, the load capacitor with capacitance $C_{lp}$ is connected in parallel with one, two, or three quartz crystals. The load capacitance reduces the impedance, as shown in Figure 1. By using Equation (3), the impedance of the load capacitor can be expressed as

(7) $$\frac{1}{j\omega C_{lp}}=\frac{2\sqrt{LC}}{j\Omega_{r}C_{lp}}\;.$$

The impedance of one quartz crystal in parallel with the load capacitor (${\bar{Z}}_{1QCl}\left(\Omega_{r}\right)$) can now be expressed as

(8) $${\bar{Z}}_{1QCl}\left(\Omega_{r}\right)=\frac{\bar{Z}\left(\Omega_{r}\right)\;\frac{2\sqrt{LC}}{j\Omega_{r}C_{lp}}}{\bar{Z}\left(\Omega_{r}\right)+\frac{2\sqrt{LC}}{j\Omega_{r}C_{lp}}}\;\;,\;$$

where $\bar{Z}\left(\Omega_{r}\right)$ is given by Equation (4). For two identical crystals in parallel and parallel to $C_{lp}$, the impedance ${\bar{Z}}_{2QCl}\left(\Omega_{r}\right)$ is given as

(9) $${\bar{Z}}_{2QCl}\left(\Omega_{r}\right)=\frac{\frac{\bar{Z}\left(\Omega_{r}\right)}{2}\frac{2\sqrt{LC}}{j\Omega_{r}C_{lp}}}{\frac{\bar{Z}\left(\Omega_{r}\right)}{2}+\frac{2\sqrt{LC}}{j\Omega_{r}C_{lp}}}\;,\;$$

while for three crystals in parallel with $C_{lp}$ we have

(10) $${\bar{Z}}_{3QCl}\left(\Omega_{r}\right)=\frac{\frac{\bar{Z}\left(\Omega_{r}\right)}{3}\frac{2\sqrt{LC}}{j\Omega_{r}C_{lp}}}{\frac{\bar{Z}\left(\Omega_{r}\right)}{3}+\frac{2\sqrt{LC}}{j\Omega_{r}C_{lp}}}\;.$$

While having two or three crystals in parallel has no effect on the resonant frequency (only the impedance is reduced), we see that the addition of the load capacitance will affect the antiresonant frequency as well, while the resonant frequency remains $f_{0}$. The resonant and antiresonant frequencies are found by setting the imaginary part of the impedance (Equation (4)) to zero. By neglecting the effect of R (R = 0) it is straightforward to find new antiresonant frequencies for one, two, or three crystals connected in parallel. Because of the parallel load capacitance, the antiresonant frequency of the crystal is pulled to frequency $f_{lp}$ [7,33]:

(11) $$f_{lp}=f_{0}\sqrt{1+\frac{C}{C_{0}+C_{lp}}}\;.$$

When two crystals are connected in parallel, the new resonant frequency ($f_{lp2}$) is

(12) $$f_{lp2}=f_{0}\sqrt{1+\frac{2C}{2C_{0}+C_{lp}}}\;,\;$$

where we have a new shunt capacitance, which is the sum of the shunt capacitances of both quartz crystals [32]. We have assumed that the quartz crystals are identical; thus, the shunt capacitance is $2C_{0}$.

When three crystals are connected in series, the shunt capacitances of all three of them affect the resonant frequency $f_{l3}$, which is thus given by

(13) $$f_{lp3}=f_{0}\sqrt{1+\frac{3C}{3C_{0}+C_{lp}}}\;,\;$$

where $3C_{0}$ is the shunt capacitance of the three equal quartz crystals.

We define the frequency pulling range ($\Delta f_{lp}$) for one crystal between two load capacitance values $C_{lp1}$ and $C_{lp2}$ as

(14) $$\Delta f_{lp}=f_{0}\left[\sqrt{1+\frac{C}{C_{0}+C_{lp1}}}-\sqrt{1+\frac{C}{C_{0}+C_{lp2}}}\right].$$

Thus, when three crystals are connected in parallel, the frequency pulling range ($\Delta f_{lp3}$) is

(15) $$\Delta f_{lp3}=f_{0}\left[\sqrt{1+\frac{3C}{3C_{0}+C_{lp1}}}-\sqrt{1+\frac{3C}{3C_{0}+C_{lp2}}}\right].$$

From Figure 1 we see that the impedance circle is reduced most when we have only one crystal in parallel with the load capacitance. The relative reduction of the impedance circle is much lower when we have two or three crystals in parallel. This means that the impedance conditions will be less affected by the load capacitance if we have multiple crystals in parallel. The multiple feedback loops in oscillators ease the oscillation of the crystals, which results in the increase in the pulling range [6].

**Figure 2:**
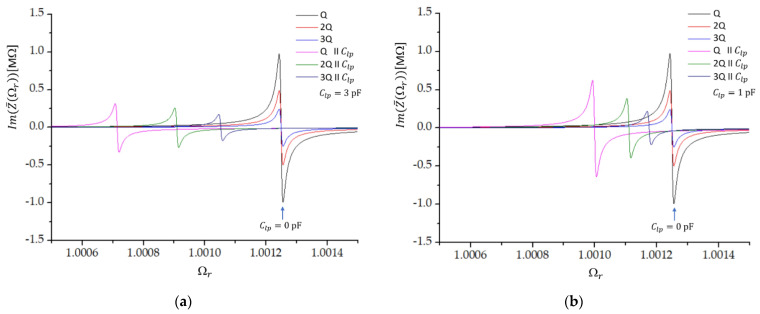
The imaginary part of impedance as a function of the frequency ratio $\Omega_{r}$ close to the antiresonant frequency of a single crystal ($\Omega_{r}=1.0012499$ ). The dependencies are shown for one (Q), two (2Q), and three (3Q) crystals in series with and without the addition of the load capacitor with capacitance (**a**) $C_{lp}=1\;\mathrm{pF}$ and (**b**) $C_{lp}=3\;\mathrm{pF}$.

Figure 2 shows the imaginary part of the impedance as a function of the frequency ratio $\Omega_{r}$ when the load capacitor with capacitance $C_{lp1}=1\;\mathrm{pF}$ is connected in parallel to one (Q), two (2Q), or three (3Q) crystals in parallel. Without the load capacitance, all three crystals have the same antiresonant frequency $1.0012499\;f_{0}$; only the amplitude is changed. When the load capacitor is added, the change in the resonant frequency as well as the reduction in the magnitude of the imaginary part of impedance are the largest in the case of one crystal and the lowest in the case of three crystals in parallel. We also see that the shift in the resonant frequency increases when the load capacitance is increased, while the magnitude of the imaginary part of impedance reduces with increasing value of $C_{lp}$ (Figure 2b).

## 3. Experimental Setup

Several oscillator electronic circuits have been investigated so far [1,33,38,39,40,41,42]. Their common feature is a problem with the stability of the crystal oscillation, the temperature effect on crystal oscillation, ageing of the crystal as well as other elements in the circuit, and the effect of parasitic impedances. We constructed an experimental circuit that reduces all the above-mentioned effects.

**Figure 3:**
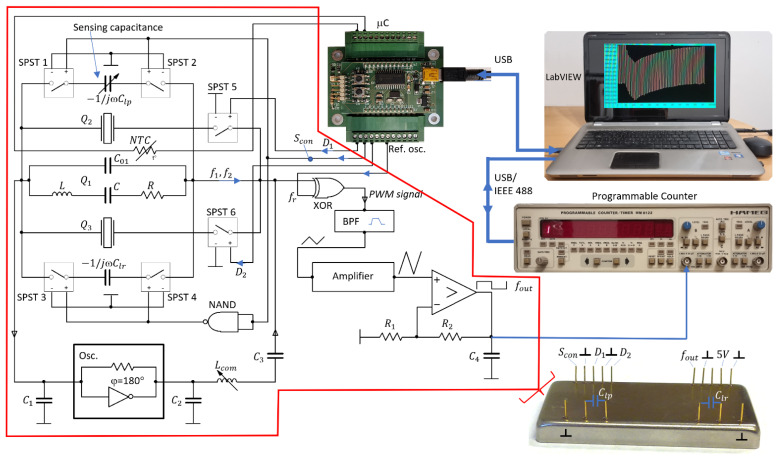
Experimental setup for switching one, two, or three crystals ($Q_{1}$, $Q_{2}$, $Q_{3}$ ) connected in parallel and for switching of the reference ($-1/j\omega C_{lr}$) and measured ($-1/j\omega C_{lp}$) reactance. For switching the crystals and realization of the switching method, a microcontroller is used. For measuring the frequency, a programable counter and LabVIEW software are used. A prototype of a low value reactance sensor includes the red-encircled elements.

The experimental setup is shown in Figure 3. Two additional quartz crystals, $Q_{2}$ and $Q_{3}$, and an element to switch these crystals, are added to the quartz crystal oscillator. The second and third quartz crystal are connected to the first crystal by signals $D_{1}$ and $D_{2}$ through SPST (Single Pole Single Throw) switches; in this way, the parasitic capacitances are always the same.

This circuit enables an accurate analysis of the operation conditions of various connection of quartz crystals. The low-value reactance transducer consists of a modified oscillator circuit with one, two, and three quartz crystals, sensing reactance, and a switching part. The novelty in this approach is the use of a specific symmetrical switching mode quartz oscillator, in which multiple quartz crystals are connected in parallel, and in parallel to them, we connect two capacitors with low value reactances $-1/j\omega C_{lp}$ and $-1/j\omega C_{lr}$, the first one being adjustable. The reactances are connected alternately with the help of the SPST 1–4 switches and enable a significant reduction in the influence of parasitic impedances on frequency change because of the symmetry of the circuit and because at any of the reactances the same combination of crystals is used. The capacitance $C_{lp}$ is a load capacitance, which enables a highly sensitive capacitance–frequency conversion at a simultaneous compensation of quartz crystals. $C_{lr}$ is a reference capacitance by which we achieve impedance conditions for a parallel connection of crystals and conditions for oscillator operation to be the same as in the case when $C_{lp}$ is connected. The switching of an oscillator, which is switching between frequencies $f_{1}$ and $f_{2}$, is realized with the help of a control digital signal $S_{con}$ (values 1 and 0) and a NAND gate, which produces an output, which is false only if both its inputs are true (Figure 3) [43]. A variable inductivity, $L_{com}$, is used to fine-tune both frequencies $f_{1}$ and $f_{2}$ for reactances $-1/j\omega C_{lp}$ and $-1/j\omega C_{lr}$ at a certain frequency $f_{r}$, and to set the sensitivity of the sensor [28,44,45,46,47]. A pulse-wide modulated signal corresponding to the frequency difference between the frequency $f_{1}$ and reference frequency $f_{r}$ (from reference oscilator) or the difference between the frequency $f_{2}$ and reference frequency enters the BPF (Band Pass Filter) [48,49,50]. If the frequencies $f_{1}$ and $f_{2}$ (in our case) equal approximately 4 MHz and are a few kHz different from the frequency $f_{r}$, then these two frequencies are converted (depending on the signal $S_{con}$) to the range between 4 and 100 kHz at the output of BPF. The upper frequency of BPF is 1 MHz and the lower one 20 Hz. A triangular signal obtained at the output of the BPF filter, which depends on $L_{com}$ and $f_{r}$ and the initially set frequency of 4 kHz, is amplified by an amplifier to ease the transformation into a rectangular signal. The obtained rectangular signal is not yet temperature compensated. The temperature compensation is attained when the two sequential output frequencies are subtracted. Temperature compensations are also effects of the stray capacitance, which affect both frequencies $f_{1}$ and $f_{2}$. The output signal frequency $f_{out}$ contains both frequencies synchronically, depending on the switching frequency $f_{con}=20\;\mathrm{Hz}$ (one measurement cycle is 50 ms). Capacitances $C_{1}$ in $C_{2}$ serve to suppress the spurious responses of the quartz crystals to avoid crystal oscillation at higher frequencies [8,9,33].

A prototype of the multiple quartz sensor in the SMD technology on an ${\mathrm{Al}}_{2}O_{3}$ PCB basis is shown in Figure 3. At the front side of the housing, the converter has pins for $C_{lp}$ and $C_{lr}$. For specific industrial applications, the refence capacitor can also be incorporated inside the housing. At the back side of the housing, there are pins for a supply voltage, $S_{con}$, $D_{1}$ and $D_{2}$ signals, and an output frequency, $f_{out}$. The main advantage of such a construction is that it allows for a connection of the capacitance-sensitive elements to these pins with very low additional parasitic capacitances, and even these are—when the switching method is used—reduced to a minimum.

The circuit with a switching mode operation, shown in Figure 3, achieves the temperature compensation of a single quartz crystal unit in the following way. When both reactances, $-1/j\omega C_{lp}$ and $-1/j\omega C_{lr}$, are almost the same, then the two frequencies $f_{1}\left(S_{con}\right)$ and $f_{2}\left(\bar{S_{con}}\right)$ are also almost the same at the signal states $S_{con}=1$ and $\bar{S_{con}}=0$, respectively, and depend on the quartz crystal resonant frequency, $f_{0}$, and changes of this frequency due to the quartz crystals’ temperature characteristics ($\Delta f_{0}\left(T\right)$); the variation in $L_{com}$ ($\Delta f_{0}\left(L_{com}\right)$); the inequality of $C_{lp}$ and $C_{lr}$; and on the quartz crystals’ ageing $\Delta f_{0}\left(t\right)$. On the other hand, when reactances $-1/j\omega C_{lp}$ and $-1/j\omega C_{lr}$ differ by approximately 0.1%, the frequencies $f_{1}$ and $f_{2}$ already differ by the order of kHz. The output frequency ($f_{out}$) is the difference between the frequency $f_{1}$ and the reference frequency $f_{r}$ ($f_{out1}=f_{1}-f_{r}$); similarly, $f_{out2}=f_{2}-f_{r}$. If we calculate the difference between the two output frequencies, $f_{out1}-f_{out2}$, $\Delta f_{0}\left(T\right)$, $\Delta f_{0}\left(t\right)$, and $\Delta f_{0}\left(L_{com}\right)$ are well compensated if the temperature variations are slower than ${1\;}^{{}^{\circ}}C/s$ because only one temperature quartz characteristic is involved. In a similar way, temperature variations in the reference frequency $\Delta f_{r}\left(T\right)$ are compensated. The output frequencies $f_{out1}$ and $f_{out2}$, corresponding to the two logical states $S_{con}$ and $\bar{S_{con}}$, are measured by a programmable counter and LabVIEW and can be expressed as

(16) $$f_{out1}=f_{0}+\Delta f_{0}\left(T_{1}\right)+\Delta f_{0}\left(t_{1}\right)+\Delta f_{0}\left(L_{com}\right)+\Delta f_{0}\left(C_{lp}\right)-(f_{r}\left(T_{1}\right)+\Delta f_{r}\left(T_{1}\right))\pm \Delta f_{er1}\left(t_{1}\right)$$

and

(17) $$f_{out2}=f_{0}+\Delta f_{0}\left(T_{2}\right)+\Delta f_{0}\left(t_{2}\right)+\Delta f_{0}\left(L_{com}\right)+\Delta f_{0}\left(C_{lr}\right)-(f_{r}\left(T_{2}\right)+\Delta f_{r}\left(T_{2}\right))\pm \Delta f_{er2}\left(t_{2}\right),$$

where $\Delta f_{0}\left(C_{lp}\right)$ and $\Delta f_{0}\left(C_{lr}\right)$ are shifts in the resonant frequency due to the measured and reference capacitance, respectively, and $\Delta f_{er1}$ and $\Delta f_{er2}$ are the measurement errors of the programmable counter at measuring times $t_{1}$ and $t_{2}$, respectively. $T_{1}$ and $T_{2}$ are temperatures at times $t_{1}$ and $t_{2}$, respectively. If the signal switching is of the order of ms, one can assume that $T_{1}≅T_{2}$ and $t_{1}≅t_{2}$; thus, the frequency shifts due to aging are the same and so are the frequency shifts $\Delta f_{0}\left(T\right)$ due to the temperature. The temperature-compensated final output frequency ($\Delta f_{TC_{out}}$) is

(18) $$\Delta f_{TC_{out}}\left(C_{lr},\;C_{lp}\right)=\Delta f_{0}\left(C_{lr}\right)-\Delta f_{0}\left(C_{lp}\right)\pm \left(\Delta f_{er2}\left(t_{2}\right)+\Delta f_{er1}\left(t_{1}\right)\right).\;$$

What is left, is the temperature variation due to the changes in the measured reactance, $-1/j\omega C_{lp}$, and the initial setting of the reference reactance $-1/j\omega C_{lr}$ and the counter measuring error at two sequential measurements of frequencies $f_{out1}$ and $f_{out2}$, at times $t_{1}$ and $t_{2}$, respectively.

In Figure 3, the quartz crystal $Q_{1}$ is presented by an equivalent electric circuit. Its stray capacitance, $C_{01}$, includes the pin-to-pin input and output capacitances (parasitic capacitances). The typical value of the stray capacitance, $C_{0}$, is between 2.5 pF and 7 pF. By an additional influence on this stray capacitance (and by this on the equivalent circuit) through the parallel load capacitance, one can affect the frequency of the stable quartz crystal oscillator such that the oscillator acts as a capacitive-frequency transducer whose resolution is in the sub aF range.

With the compensation of $C_{0}$ by a series inductivity $L_{com}$, as described in [5,8,9,25], one can obtain an almost linear dependence of frequency $f_{1}$ on $C_{lp}$ close to the quartz resonant frequency. When three crystals are connected in parallel, $L_{com}$ is used to compensate the sum of all three shunt capacitances $(C_{01}+C_{02}+C_{03}$), and the frequencies $f_{1}$ and $f_{2}$ for both signal states $S_{con}$ are

(19) $$f_{1}\left(S_{con},\;S_{v},\;C_{lp}\;\right)=\frac{1+\frac{C}{2\left(C_{01}+C_{02}+C_{03}+C_{lp}-\frac{1}{\omega_{0}{}^{2}S_{v}L_{com}}\right)}}{2\pi \sqrt{LC}}+\Delta f_{0}\left(T_{1}\right)\pm \Delta f_{0}\left(t_{1}\right)$$

and

(20) $$f_{2}\left(\bar{S_{con}},\;S_{v},\;C_{lr}\;\right)=\frac{1+\frac{C}{2\left(C_{01}+C_{02}+C_{03}+C_{lr}-\frac{1}{\omega_{0}{}^{2}S_{v}L_{com}}\right)}}{2\pi \sqrt{LC}}+\Delta f_{0}\left(T_{2}\right)\pm \Delta f_{0}\left(t_{2}\right)\;,$$

where $S_{v}$ is a compensation factor, which depends on the compensation inductance and capacitances $C_{01}$, $C_{02}$, and $C_{03}$ [51].

Frequencies $f_{1}$ and $f_{2}$ (Equations (19) and (20)) depend on the switching state ($S_{con}$ or $\bar{S_{con}}$), chosen sensitivity $S_{v}$, magnitude of the compensated shunt capacitance ($C_{01}+C_{02}+C_{03}$), and on the magnitude of the measured ($C_{lp}$) and reference ($C_{lr}$) capacitance. Both frequencies depend also on the temperature change during the two switches and on the aging of the crystals. When the switching rate of the signal is high (of the order of ms), in two sequential switches of the signal and two sequential measurements of the corresponding frequencies $f_{1}$ and $f_{2}$, followed by a subtraction of these two frequencies, the temperature variation and ageing are practically completely compensated.

The switching between $S_{con}$ and $\bar{S_{con}}$ signals also compensates the auxiliary, reference frequency $f_{r}$, and consequently also its temperature instability. This results in the frequency difference $\Delta f_{out}$ representing the temperature-compensated value of the output frequency, depending solely on the difference between $\Delta C_{lp}$ and $\Delta C_{lr}$:

(21) $$\Delta f_{out}\left(S_{v},\;C_{lp},C_{lr}\right)=\;f_{1}\left(S_{con},\;S_{v},\;C_{lp}\;\right)-f_{2}\left(\bar{S_{con}},\;S_{v},\;C_{lr}\;\right)\pm \left(\Delta f_{er1}\left(t_{1}\right)+\Delta f_{er2}\left(t_{2}\right)\right).$$

**Figure 4:**
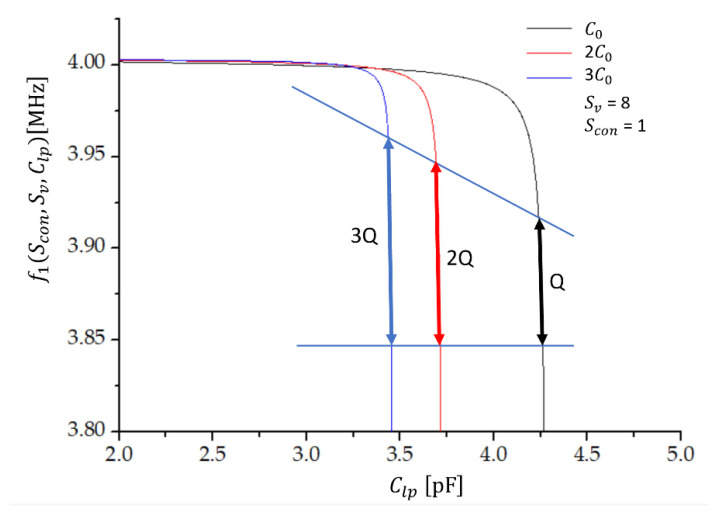
The frequency ($f_{1})$ and load capacitance ($C_{lp})$ characteristics for one (Q), two (2Q), and three (3Q) quartz capacitors connected in parallel, with $L_{com}=70$ μH, $S_{v}=8$, $S_{con}$ = 1, and T=25 °C. $C_{0}$ is the shunt capacitance of one quartz crystal.

Figure 4 shows the oscillator’s frequency characteristics $f_{1}\left(S_{con},\;S_{v},\;C_{lp}\;\right)$ (Equation (19)) as a function of the load capacitance $C_{lp}$ for a different number of crystals connected in parallel at the compensation factor $S_{v}=8$ and for the state $S_{con}$= 1 (T=25 °C). The coloured arrows denote the regions of approximately linear dependence of $f_{1}$ on $C_{lp}$. We see that the sensitivity ($\Delta f_{1}/\Delta C_{lp}$) is predicted to be the highest in the case of three quartz crystals in parallel (blue line). At the chosen parameters, the sensitivity is 1.3 kHz/fF in the range of 10 fF around the value of $C_{lp}$ of the order of pF, by requiring a linearity of 0.2% of the capacitance–frequency characteristics.

## 4. Results and Discussion

To measure the sensitivity and capacitance sensing range, the experimental setup shown in Figure 3 was used. As already explained, this setup ensures that the parasitic capacitances, inductances, and impedances are very low, and, in addition, due to the switching method they have practically no influence on the measured output frequency, which enables good repeatability of the experimental results. By the symmetry of the circuit, equal conditions for capacitances $C_{lp}$ and $C_{lr}$ are achieved, which is also crucial for the success of the method.

The step variation in $C_{lp}$ was achieved by laser trim capacitors [52,53,54,55,56]. For this measurement, the capacitors with capacitance $C_{lp}$ and coils with inductance $L_{com}$, both with a tolerance of 0.2 %, were selected [47] by measurements with the Keithley 4200A-SCS parameter analyser equipped with a 4215 CVU (high-resolution capacitance–voltage unit), the resolution of which is 1 aF in the measuring range 10 aF do 10 pF. The load capacitance $C_{lp}$ varied from 3.300 to 4.300 pF in steps of 2 fF with a 1 aF resolution in the region where sensitivity is the highest (regions denoted by arrows in Figure 4). A capacitive matrix, described in [29,57,58], where it was used to calibrate a 14-bit capacitive SAR register, was used to set the capacitance with an 8-bit accuracy in the region of 336 fF.

The capacitance of the reference capacitor was set to $C_{lr}=$ 3.450 pF. The quartz crystal parameters were C=10 fF, L=158.314 mH, $C_{0}=4\;\mathrm{pF}$, and R=10 Ω, measured with a HP4194a impedance/gain phase analyser. At these parameters, the resonant frequency is $f_{0}=4\;\mathrm{MHz}$.

The sensitivity ($\Delta f_{out}/\Delta C_{lp}$) and region of capacitance over which the response is linear (the capacitance sensing range $\Delta C_{lp}$), requiring a linearity within 0.2%, are collected in Table 1 for different compensation factors and compensation inductivities. The compensation principle presented in [51] was used. Measurements were performed at temperature $T={25\;}^{{}^{\circ}}C$, stabilised to $\pm {0.2\;}^{{}^{\circ}}C$. The results show that the sensitivity increases and the capacitance sensing range decreases if (i) more crystals are connected in parallel; (ii) the compensation factor increases; and (iii) compensation inductivity increases.

**Figure 5:**
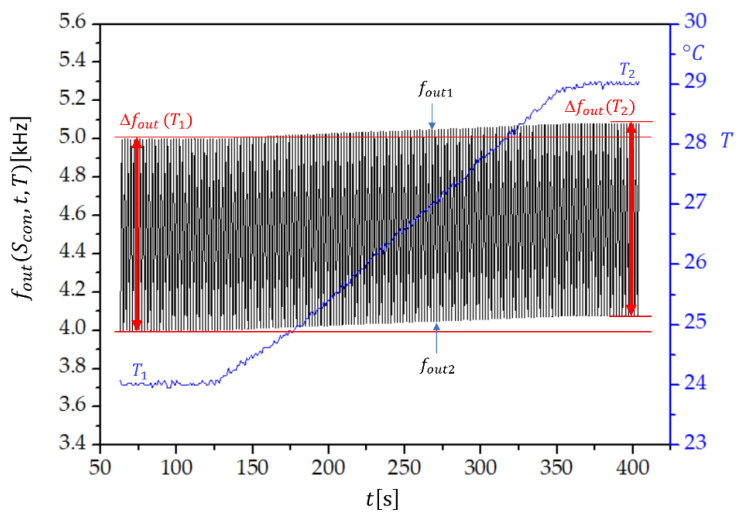
Extended temperature dynamic frequency stability for $f_{out1}=f_{1}\left(S_{con}\right)-f_{r}$ and $f_{out2}=f_{2}\left(\bar{S_{con}}\right)-f_{r}$ if three quartz crystals are connected in parallel, where $S_{v}=8$, $L_{com}=70\;\mu H$, $C_{lp}=3.459\;\mathrm{pF}$, and $C_{lr}=3.450\;\mathrm{pF}$, and the quartz crystal parameters C=10 fF, L=158.314 mH, $C_{0}=4\;\mathrm{pF}$, and R=10  Ω. $T_{1}$ and $T_{2}$ are the initial and final temperatures, respectively, and $\Delta f_{out}\left(T_{1}\right)$ and $\Delta f_{out}\left(T_{2}\right)$ are the corresponding frequency differences $\Delta f_{out}=f_{out1}-f_{out2}$.

Figure 5 shows the switching mode extended dynamic stability, i.e., the frequency change of $f_{out}\left(S_{con},t,T\right)$, if the temperature of the sensor increases from $T_{1}={24\;}^{{}^{\circ}}C$ to $T_{2}={29\;}^{{}^{\circ}}C$ in the time span of 400 s. We see that the temperature change has an influence on $f_{out1}=f_{1}\left(S_{con}\right)-f_{r}$ and $f_{out2}=f_{2}\left(\bar{S_{con}}\right)-f_{r}$. However, $\Delta f_{out}=f_{out1}-f_{out2}=f_{1}\left(S_{con}\right)-f_{2}\left(\bar{S_{con}}\right)$ remains the same, which proves that the temperature influence on $f_{1}$, $f_{2}$, and $f_{r}$ is compensated. Similarly, the influence of temperature change on the frequency measurement error produced by the frequency counter is significantly reduced. The dynamic temperature change of both frequencies is approximately the same. The temperature dependence of $\Delta f_{out}\left(T\left(t\right)\right)$, shown in Figure 5, is universal for all the compensation factors. Its magnitude is set by the value of $C_{lr}$.

**Figure 6:**
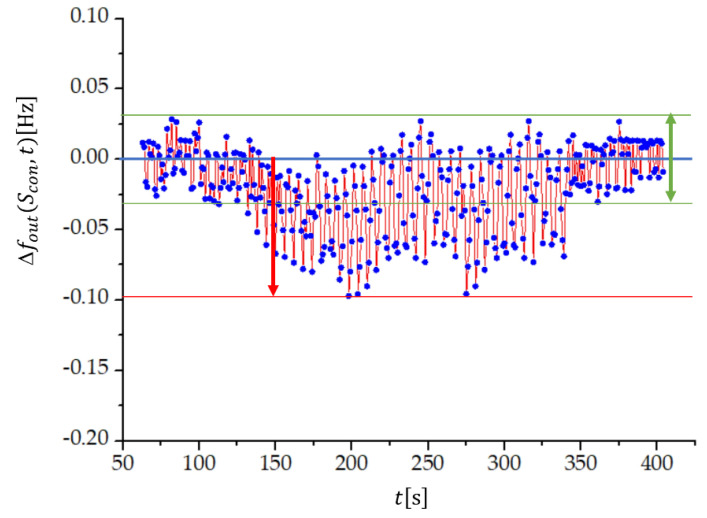
Output frequency dynamic error, $\Delta f_{out}=f_{1}-f_{2}\pm \left(\Delta f_{1}+\Delta f_{2}\right)$, measured at $f_{1}-f_{2}=0$ during the change in temperature from 24 °C to 29 °C, as shown in Figure 5, for three quartz crystals connected in parallel, where $S_{v}=8$, $L_{com}=70\;\mu H$, $C_{lp}=3.459\;\mathrm{pF}$, and $C_{lr}=3.450\;\mathrm{pF}$, and the quartz crystal parameters C=10 fF, L=158.314 mH, $C_{0}=4\;\mathrm{pF}$, and R=10  Ω.

By including also the uncertainties in the measured frequencies, $\Delta f_{out}$ equals $\Delta f_{out}=f_{1}\left(S_{con}\right)-f_{2}\left(\bar{S_{con}}\right)\pm \left(\Delta f_{1}+\Delta f_{2}\right)$, where $\Delta f_{1}$ and $\Delta f_{2}$ are the uncertainties of the measured frequency differences $f_{out1}$ and $f_{out2}$. The frequency stability at temperature variation can thus be conveniently studied at $f_{1}=f_{2}$, because in this case $\Delta f_{out}=\pm \left(\Delta f_{1}+\Delta f_{2}\right)$. Measurements at $S_{v}=8$ and $L_{com}=70\;\mu H$ are shown in Figure 6, but the characteristic is universal for all compensation factors and compensation inductances. We see that the uncertainty of the frequency difference is lower if the temperature is constant (green arrow in Figure 6) and it is the same both at lower and higher temperature. When temperature is changing, the uncertainty increases (red arrow), but its value remains lower than 0.1 Hz. In addition, Figure 6 illustrates the temperature compensation of the quartz crystal’s natural temperature characteristics from Figure 5.

Now we can return to results in Table 1 and argue that the method enables measurements of changes in capacitance in a femtofarad range with a zeptofarad sensitivity, because a change in frequency as low as 0.1 Hz can be measured if the temperature is changing (Figure 6), and even lower (0.03 Hz) if the temperature is kept constant. The method is most sensitive if we have three quartz crystals, with the maximum compensation factor, $S_{v}=16$, at maximum compensation inductance, $L_{com}=1\;\mathrm{mH}.$ From Table 1 we see that, in this case, the sensitivity is 32.500 kHz/fF in a range of $\Delta C_{lp}=4\;\mathrm{fF}$. Thus, under a dynamic temperature condition one can detect changes in capacitance equal to 0.1/32,500 fF, which equals approximately 3 zF. At two quartz crystals, and the maximum $S_{v}$ and $L_{com}$, we have 0.1/13,000 fF, which is approximately 8 zF, but over a 2.5-times wider capacitance sensing range, $\Delta C_{lp}$, than in the case of three quartz crystals. If only one quartz crystal is used, then changes in capacitance 0.1/3612 fF, i.e., 28 zF, can be detected. We see that by connecting three quartz crystals in parallel, the sensitivity increases by an order of magnitude, while the capacitance sensing range reduces by an order of magnitude. Compared to other methods to detect low capacitance changes, which are not based on quartz crystals (an overview over these methods can be found in [26,29]), the here-proposed method offers a 20 to 100 times higher sensitivity.

## 5. Conclusions

We have presented a measuring method that employs several quartz crystals connected in parallel to measure small capacitance changes with a high accuracy and with the simultaneous compensation of several effects, especially compensation of the eigen frequency–temperature characteristics of the quartz crystals. This compensation is essential when the sensing (load) capacitance changes are in the fF or aF region. The presented method opens new applications in physics, chemistry, pharmacy, mechatronics, biosensor technology, and in all industrial applications that demand high-quality production.

The experimental circuit shown in Figure 3 was used to reduce the effect of parasitic impedances, the temperature characteristics of the quartz crystals, the influence of dynamic changes in the environmental temperature, and the ageing of crystals and other elements in the circuit. The method also reduces the start-up time of the oscillator down to 1 min.

To detect very low changes in capacitance of the order of fF and aF, it is essential to compensate the parasitic capacitances, which was achieved by the switching mode method and the symmetry of the oscillator circuit. When frequencies $f_{1}$ and $f_{2}$, obtained at two switches, are subtracted, we also subtract the effects of all the parasitic impedances. To have these effects as equal as possible at the two sequential switches, we used SPST 1–5 sequential switches. The circuit is made on an aluminium oxide ${\mathrm{Al}}_{2}O_{3}$ PCB basis, which has a very low coefficient of thermal expansion ($6-7.5\times 10^{-6}\;K^{-1}$). The reference capacitor has a capacitance $C_{lr}$, which is approximately the same as the capacitance of the sensing (load) capacitance $C_{lp}$. In this way, equal conditions for crystals are achieved at both switches. The switching method also reduces the effect of the short- and long-term stability of the quartz oscillator. Next to the compensation of the temperature characteristics, the switching method also compensates the temperature instability of the reference oscillator because this effect is also subtracted after two sequential switches.

We have used AT-cut crystals with a $0^{\prime }$-cut angle (the crystal’s x cut axis is tilted by 35°15′ with respect to the optic axis), because these crystals have the lowest dependence of frequency on temperature (±3 ppm in a temperature region $0–{50\;}^{{}^{\circ}}C$). The frequency $f_{0}=4\;\mathrm{MHz}$ was selected due to a higher *Q* value (≅80k). The switching method enables compensation of the AT-cut crystal temperature characteristics below 0.01 Hz in the temperature range $0–{50\;}^{{}^{\circ}}C$. It was shown that this enables a zeptofarad resolution.

The main results, presented in Table 1, show that a connection of two or three quartz crystals in parallel significantly increase the frequency–capacitance sensitivity, providing that the quartz crystals oscillate with the same frequency in the oscillator. The load (sensible) capacitance is connected in parallel to the quartz crystals. By increasing the number of quartz crystals connected in parallel, the sensitivity increases and the capacitance sensing range decreases. The sensitivity increases also by increasing the compensation factor, defined by the compensation inductance. For the performed measurements, the highest sensitivity, $\Delta f_{out}/\Delta C_{lp}=32.500\;\mathrm{kHz}/\mathrm{fF}$, was achieved at a compensation factor $S_{v}=16$ ($L_{com}=1\;\mathrm{mH}$) and three quartz crystals in parallel. The capacitance sensing range in this case was 4 fF and the sensitivity 3 zF under a dynamic temperature variation. If temperature is kept constant, the sensitivity is even higher, approximately 1 zF.

To sum up, the advantages of the proposed measurement method based on the use of multiple quartz crystals connected in parallel are increased sensitivity; measuring range in the fF region with the resolution in the zF region; a sensitivity of 32 kHz/fF in a sensing range of 4 fF (for three quartz crystals in parallel, the maximum compensation factor and compensation inductance); high linearity of the frequency–capacitance characteristics within the sensing region; compensation of the temperature nonlinear characteristics of the crystal and oscillator elements; compensation of the ageing of crystals and other elements in the circuit; compensation of parasitic impedances; compensation of changes in the reference frequency; and compensation of the changes in the applied voltage. The output frequency is in the range of 4–100 kHz, which is the proper region for further processing of the signal by a microcontroller.

## Figures and Tables

**Table 1:** The sensitivity, $\Delta f_{out}/\Delta C_{lp}$, and capacitance sensing range, $\Delta C_{lp}$, at different compensation factors $S_{v}$ and compensation inductivities $L_{com}$ for one (Q), two (2Q), and three (3Q) crystals in parallel (C=10 fF, L=158.314 mH, $C_{0}=4\;\mathrm{pF}$, R=10  Ω) measured at *T* = 25 °C.

		Q		2Q		3Q	
$S_{v}$	$L_{com}\;\left(\mu H\right)$	$\Delta f_{out}/\Delta C_{lp}$ (kHz/fF)	$\Delta C_{lp}\;\left(fF\right)$	$\Delta f_{out}/\Delta C_{lp}$ (kHz/fF)	$\Delta C_{lp}\;\left(fF\right)$	$\Delta f_{out}/\Delta C_{lp}$ (kHz/fF)	$\Delta C_{lp}\;\left(fF\right)$
1	4	0.387	336	0.532	244	0.687	189
2	7	0.479	271	0.812	160	1.287	101
4	10	0.599	217	1.203	108	2.131	61
6	40	0.516	252	0.956	136	1.529	85
8	70	0.807	161	1.857	70	3.823	34
10	100	1.214	107	3.421	38	6.842	19
12	400	0.935	139	2.364	55	4.815	27
14	700	1.969	66	6.500	20	13.001	10
16	1000	3.612	36	13.000	10	32.500	4

## Data Availability

All the additional data is available from the author upon reasonable request.
